# Challenges of a community based pragmatic, randomised controlled trial of weight loss maintenance

**DOI:** 10.1186/s13104-015-1791-7

**Published:** 2015-12-18

**Authors:** Elizabeth Randell, Rachel McNamara, Christine Shaw, Aude Espinasse, Sharon Anne Simpson

**Affiliations:** South East Wales Trials Unit, Heath Park, Cardiff University, Cardiff, Wales UK; MRC/CSO Social and Public Health Sciences Unit, University of Glasgow, 200 Renfield Street, Glasgow, G2 3QB Scotland UK

**Keywords:** Randomised controlled trial, Recruitment, Challenges, Barriers, Complex intervention, Community

## Abstract

**Background:**

Randomised controlled trials (RCTs) have a reputation for being inherently difficult to deliver as planned and often face unforeseen challenges and delays, particularly in relation to organisational and governance difficulties, participant interest, constraints due to allocation of costs, local investigator interest and lengthy bureaucracy. Recruitment is often difficult and the challenges faced often impact on the cost and delivery of a successful trial within the funded period. This paper reflects upon the challenges faced in delivering a pragmatic RCT of weight loss maintenance in a community setting and suggests some potential solutions.

**Methods:**

The weight loss maintenance in adults trial aimed to evaluate the impact of a 12 month, individually tailored weight maintenance intervention on BMI 3 years from randomisation. Participants were recruited primarily from participant identification centres (PICs)—GP surgeries, exercise on referral schemes and slimming world. The intervention was delivered in community settings. A recruitment strategy implementation plan was drafted to address and monitor poor recruitment.

**Results:**

Delays in opening and recruitment were experienced early on. Some were beyond the control of the study team such as; disagreement over allocation of national health service costs and PIC classification as well as difficulties in securing support from research networks. That the intervention was delivered in community settings was often at the root of these issues. Key items to address at the design stage of future trials include feasibility of eligibility criteria. The most effective element of the recruitment implementation plan was to refocus sources of recruitment and target only those who could fulfil the eligibility criteria immediately.

**Conclusions:**

Learnings from this trial should be kept in mind by those designing similar studies in the future. Considering potential governance, cost and research network support implications at the design stage of pragmatic trials of any community-based complex intervention is paramount. The appropriateness and viability of inclusion criteria also require careful consideration as does use of a targeted advertising strategy.

*Trial registration*: ISRCTN35774128, 12/01/2010

## Background

Randomised controlled trials (RCTs) are generally considered the gold standard for evaluating healthcare interventions [1]. However, they also have the reputation for being inherently difficult to run as planned and face numerous challenges and delays, particularly in relation to governance processes. Recruitment is often much slower and more difficult than anticipated with unforeseen difficulties leaving many trials unable to achieve their planned targets within the funded period [2]. One systematic review suggests that as many as 50 % of trials fail to recruit to target and of those that are able to do so, 50 % exceed their planned recruitment period [3]. The impact and consequences of poor recruitment have the potential to leave a trial underpowered and thus unable to answer the research questions posed.

Evidence from a systematic review and previous research highlight particular difficulties commonly faced in trials when asking clinicians to recruit, issues include; remembering to approach potential participants, complicated recruitment criteria, difficulty explaining the study, time constraints and lack of staff and training [4]. However there are also issues experienced by patients which deter them from taking part, such as additional demands of the trial, concerns about data protection and consent [5]. Even when completing a feasibility study as per medical research council (MRC) guidance [6] which endeavours to assist the adoption of appropriate methods, it is not always possible to avoid pitfalls nor anticipate likely problems in a future study. Although a feasibility trial may indicate a certain recruitment rate, this doesn’t necessarily reflect what might happen in a larger trial: motivation may vary between recruiters and the ability of a research team to maintain high levels of engagement with sites and support particular concerns decreases as study size increases [7]. Some acknowledged issues can be planned for but recruitment rates can of course be influenced by many factors which aren’t necessarily under the control of the investigator e.g., organisational and governance difficulties, participant interest, constraints due to allocation of costs, local investigator interest and lengthy bureaucracy [3, 8, 9].

Prolonged and convoluted approval processes have been identified as contributing factors to delays in obtaining governance approval and holding up research activities including recruitment and opening sites in new areas [10, 11]. The system of obtaining approvals is still not always consistent (for example local national health service (NHS) governance bodies often raise multiple queries relating to ethics and/or design that are global rather than local). At the time of this trial, the health research authority (HRA) had not introduced their streamlined process which intends to make this aspect of gaining approvals more coordinated. The system is still not fully operational and challenges remain. There are well documented issues relating to availability of eligible participants including the tendency to overestimate the eligible population [12].

Particular challenges are faced by community-based studies which often struggle to cope with the demands of diffuse populations, various recruitment and delivery settings and a wide variation in terms of clinicians and local practice [13]. Organisational characteristics of research teams such as previous research experience and expertise can very widely [14]. Attempts to remedy poor recruitment then have implications in terms of cost, time and staff resources. Implementing strategies to improve recruitment rates usually mean protocol changes—a process which, in itself, adds time and administrative burden. This involves decision making, documenting decision making, creating applications to REC and research and development (R&D), submission to committees, waiting for responses and then implementation of changes. Often trials will be financially curtailed in what they are able to implement as part of a revised recruitment strategy and staff may not be able to physically handle demands of the added workload.

This paper reports challenges faced in delivering the weight loss maintenance in adults (WILMA) trial. This unique trial was originally designed as a pragmatic RCT of a complex public health intervention delivered in non-NHS community settings (such as community centres) to an obese adult population who had already achieved a 5 % weight loss. This level of weight loss has been shown to be associated with improved cardiovascular disease risk factors [15]. It aimed to evaluate the impact of an individually tailored intervention on participant body mass index (BMI) 3 years from randomisation [16]. Participants were individually randomised to one of three arms: an intensive intervention arm, a less intensive intervention arm and a control arm. Those in the two experimental arms received a 12 month intervention based on motivational interviewing (MI) and self-monitoring while the control arm received an information pack and usual care. Follow-up assessments were planned for 6, 12, 24 and 36 months post-randomisation.

Despite the best efforts of the study team, recruitment targets were not achieved and in September 2012, the decision was taken by the funder to close the trial to recruitment. It was further agreed that the trial would be redesigned as a feasibility study and close completely in January 2014. The impact of this meant that a number of the planned analyses could not be completed and the initial research question could not be fully answered. This will be more fully reported in the health technology assessment programme (HTA) monograph [16]. The purpose of this paper is to describe the main challenges experienced in setting up, recruiting to and delivering the WILMA trial and to highlight practical issues that could potentially impact on the success of future similar trials of complex public health interventions delivered in community (i.e., non-NHS) settings. In addition, details of the trial recruitment strategy implementation plan will be examined in relation to impact (i.e., success), ease of implementation and cost.

## Method

Recruitment was expected to run for 18 months from October 2010 to April 2012 and the sample size calculation set a recruitment target of 950 adults (aged 18–70). Inclusion criteria were BMI (≥30 kg/m^2^) in the past 12 months and an intentional weight loss (at least 5 % of their body weight) during the same period.

Participants could enter the trial via one of two routes. Route 1 was for those who had achieved a 5 % weight loss and were able to provide independent verification of this (e.g., from a referring practitioner, slimming club record, printout from scales at chemist/supermarket). Route 2 was for those who were yet to achieve 5 % weight loss. Those in this route were invited to either: (a) attend a screening meeting with a researcher or (b) ‘self-screen’ by providing verified evidence of starting weight and subsequent 5 % loss. They were asked to contact the study team once they had achieved the 5 % target or were followed up by telephone.

A variety of sources (including some NHS settings) were used for recruitment as it was envisaged that the intervention, if successful and shown to be cost effective, could be rolled out to a wide variety of people who had lost weight using different methods. The main sources detailed in the original protocol were general practitioner (GP) practices, exercise on referral schemes (government funded schemes offering access to a supported exercise programme with the help of a specialist adviser), commercial weight loss programmes [i.e., slimming world (SW)], gyms and adverts placed in community settings. Previous experience of recruiting from these or similar sources for other studies [17–19], coupled with data obtained from contacts in a small sample of GP practices and exercise on referral data, formed the basis of our sample size calculation and estimation of recruitment timelines.

Recruitment processes were described in the study protocol from the outset [16]. Recruiters were given full training in study processes and supported by the study team as outlined in Table 1. Recruitment opened in South Wales first where governance approvals and costs were obtained more speedily.

**Table 1:** Methods of supporting recruiters

1	Training sessions	Team members provided training sessions and advice on how to approach patients
2	Study materials	Recruiters were provided with posters, flyers and ‘patient packs’ which included information sheets and pre-paid envelopes to return expressions of interest
3	Telephone contact and support	Monthly contact was made with practices in order to troubleshoot any problems and encourage staff
4	Newsletters	Quarterly newsletters were drafted to provide updates on study progress

As GP practices often record patients’ weight and it was anticipated that this would be a readily available way of evidencing weight loss for many potential participants. Nineteen practices across four health boards were recruited and set up as patient identification centres (PICs). Reimbursements were made to practices for every patient provided with postal information about the study (£5) and for every approach the GP made in person (£10) that resulted in an expression of interest being returned to the study team.

Eleven local authority exercise on referral schemes, government funded schemes offering access a supported exercise programmes, were used as their clients’ weights are routinely measured and logged. Scheme managers were supportive of the trial and encouraged their staff to participate as recruiters. Unlike GP practices, exercise on referral staff were not routinely reimbursed (i.e., via NHS costs) for identifying potential participants.

The study team also worked with SW who gave full backing to the trial and confirmed the support of their regional and local consultants. Press releases and news items were placed in the national SW magazine and on their website to draw clients’ attention to the study. The trial was also presented to consultants at regional meetings for them to take to their clients at local weekly meetings. Financial reimbursement was not available to SW consultants for their help in recruiting participants.

Recruitment via these sources was expected to open in a similar way throughout South West England and the East Midlands. The intervention was delivered in community settings by motivational interviewing practitioners (MIPs) who were recruited on a freelance basis and trained during the trial set-up phase. The study team was advised by the Department of Health (DoH) that the cost of the intervention was to be covered by the NHS as an excess treatment cost (ETCs).

All trial processes and documentation were approved by Wales Research Ethics Committee 3 and the relevant research and development committees within the NHS. In Wales, the Welsh Government body, National institute for social care and health research (NISCHR), provided a nationwide centralised process for obtaining research governance, NHS costs and research network support. In England, these were dealt with by different agencies at a local level.

## Results and discussion

### Delays in opening for recruitment

#### NHS costs

Defining and accessing NHS costs in England was extremely complex, time consuming and delayed opening to recruitment. There were difficulties in obtaining consensus on a standard treatment for obesity as well as a great deal of regional variation in the interpretation of attribution and liability of ETCs. Following huge efforts from the research team involvement from the DoH and relevant strategic health authorities, costs were only agreed by the time the trial closed to recruitment in September 2012. Discussions surrounding ETCs had been ongoing since June 2010, a period of 27 months during which GP practices did not open for recruitment in England.

Despite significant changes to guidance on attribution of NHS costs [20], there still remain a number of grey areas for interventions delivered by non-NHS staff, i.e., community public health interventions, even where these interventions would feasibly be delivered or commissioned by the NHS if proven to be effective and as such require NHS Research Ethics Committee (REC)/governance approval. Changes to commissioning arrangements in England still make it extremely difficult to recover the costs of some interventions which aren’t covered by funders. The likelihood of this impacting negatively on future trials may be relatively high as trials seek to explore new areas of research and push the boundaries of current practice. We feel it is inappropriate that issues such as these can influence the progress of this type of research to such a degree. Complex interventions often incur costs that cannot be clearly assigned and which are not easily reconciled. It should not be the case that trials are put at risk and curtailed to only working in ways that current procedures can accommodate. There needs to be more dynamic and flexible approaches to allocation of costs with a clear understanding of that process on all sides or we risk sacrificing important scientific questions in favour of those which are more bureaucratically friendly.

#### Obtaining governance approval

Additional delays arose over whether GP practices should be classified as PICs or research sites. Practices were undoubtedly acting as PICs rather than full sites—staff at these locations were not actively involved in any aspect of the research other than helping identify potential participants [21]. However, at the time of set up of the WILMA trial, PICs were a relatively new concept with regional variation in understanding what their responsibilities were. PIC classification in some regions in England was not accepted by comprehensive local research networks (CLRNs) meaning that full site approval had to be obtained in these areas. Added to the lack of resolution with regards to NHS costs, the significant delays in reaching this agreement meant that recruitment in England fell a long way behind schedule and GP practices were never opened as sites by the time the trial closed to recruitment. It is not only extremely time consuming for trial staff to resolve such issues but also very frustrating and disheartening when processes are not clear and there is variation in interpretation.

#### Research network support

As a United Kingdom clinical research network (UKCRN) portfolio trial, WILMA was designed and funded in such a way that clinical research officers/clinical studies officers from the relevant research networks supplemented the core research team. Successful delivery of this large multicentre trial was dependent on this, however there were differences—which still exist—in the infrastructure of that support between devolved nations at the time of the trial. There were also differences across England as we also found in another trial [22], specifically, the interpretation of the roles and responsibilities of network staff and their capacity to support the study. This impacted on the feasibility of delivery and cost of the study, as the only way to resolve the issue was to pay for extra staffing. Added to the other unresolved delays, recruitment could only happen in some areas via non-NHS routes, i.e., SW and through advertising. Different models of working between England and Wales meant that the trial was running to different time frames in each place.

It is important to bear in mind that there is often a large time lag between submission of an outline proposal to a major funder and the start date of a trial. Infrastructure may change during this time, making what seemed like a perfectly feasible approach at the planning stage subsequently unachievable.

### Challenges with recruitment

#### Time delays

Notwithstanding these various obstacles, the trial also faced challenges with recruitment which were magnified by the issue of not being able to open sites in England. Participant recruitment in Wales ran for 15 months from July 2011 to September 2012. In England however, recruitment was open for just four months during this period. No participants were recruited in the South West and just seven were recruited in the Midlands. Evidence on the number of people approached about the trial during this time is anecdotal as logs were poorly completed and very few were returned.

#### Eligibility criteria

It was anticipated that the majority of participants would enter the study via route 1 (as described above). However the reality was that by the end of the trial, of those 1284 EOI received, just 241 (18.7 %) were able to provide independent verification of weight loss. The vast majority (1043, 81.2 %) of those expressing an interest were not to do so and thus fell into the route 2 category (Table 2). This element of the inclusion criteria obviously had a huge impact on recruitment and created a considerable bottleneck for those wishing to enter into the study.

**Table 2:** Expressions of interest (EoI) received compared to numbers recruited

Recruiter	Route 1		Route 2		Total	
	EoI	Recruited	EoI	Recruited	EoI	Recruited
GP/nurse	91	51 (56.0 %)	830	15 (1.8 %)	921	66 (7.2 %)
SW	65	47 (72.3 %)	17	0 (0.0 %)	82	47 (57.3 %)
Exercise on referral	24	19 (79.2 %)	133	3 (2.3 %)	157	22 (14.0 %)
Other/advertising	61	34 (55.7 %)	63	1 (1.6 %)	124	35 (28.2 %)
Total	241	151 (62.7 %)	1043	19 (1.8 %)	1284	170 (13.2 %)

*GP* general practitioner, *SW* slimming world, *EoI* expression of interest

Investigations revealed a number of reasons for this. The first was being able to effectively identify potential participants. GP practices could reach a large number of individuals but unrefined database search tools and/or a lack of information to assess eligibility meant that the majority fell into route 2. For exercise on referral schemes, although many who attend were overweight or obese, they were not necessarily interested in weight loss.

It became apparent that linking in with a weight loss intervention study or weight management programme like SW, was a much more effective and resource friendly way of recruiting eligible individuals. Although a significant proportion of participants overall were recruited via GPs, there was low specificity with this resource intensive approach. Having the backing of SW headquarters and being invited to approach their clients and consultants was the most direct way of targeting potentially interested and eligible individuals.

#### PIC engagement

This was difficult especially given their remoteness from the intervention delivery. The study team invested a lot of time providing telephone, email and in-person support and encouragement to help resolve any issues creating a barrier to recruitment [20]. Notably fewer opportunistic (i.e., face-to-face) approaches were made compared to postal approaches. Reasons for this included lack of time during consultations and a need to focus on more acute issues requiring immediate management. There may also be issues about broaching the subject of obesity with patients [23]. Engagement from exercise on referral and SW staff was also generally quite low despite encouragement from management. Lack of PIC activity also increased the intended gap between training intervention practitioners and the point at which they saw participants. As a result, there was drop out of practitioners thus requiring new recruitment of staff. Training and refresher training was required thus adding to the resource burden on the team.

### Recruitment strategy implementation plan

In response to the issues being faced, the trial recruitment plan was revised and an implementation plan was drafted to describe and prioritise ways in which recruitment could be improved, expanded and monitored (see Table 3). The plan was divided into sections for maximising the impact/success of recruitment via PICs; advertising and other routes. It was prioritised according to timescale, ease of implementation with the resources available and impact based on available current research as well as our own experiences [13]. Due to time constraints and recruitment pressure, many of the recruitment strategies devised in the plan were implemented at the same time. Therefore it was not possible to gather the appropriate data in order to make a detailed analysis of the impact of each aspect of the recruitment plan. Impact has been described here based on the experience of the study team.

**Table 3:** Summary of the recruitment strategy implementation plan

Task	Priority	Actions taken
Section 1—PICs		
Expansion of recruitment territory	High	Local geographical expansion of four more PICs within the remaining recruitment timeframe
Incentives for slimming world and exercise on referral staff	High	For exercise referral staff, a £20 high street voucher for the best recruiter bi-monthly. For SW consultants, a £20 voucher for every five participants recruited per month. There was also a £20 voucher for the best SW recruiter each month (N.B. GP PICs were reimbursed via NHS support costs)
Presenting to slimming world	High	Attend SW groups to present study to clients 47 meetings attended. Attend SW regional consultants meetings. 11 meetings attended
Increased SW HQ involvement	High	Advert placed in SW magazine and email from Head of Nutrition Research at SW to consultants encouraging involvement
Increased contact with PICs	High	Monthly phone contact with all PICs (n = 75), bi-monthly PIC newsletters, repeat training sessions
Engage with slimming club on referral from GP practices in South West England	Medium	Not implemented as study closed to recruitment before opening in SW England
Monitoring PICs	Medium	Monitor poor recruiters for support. Decided against closing them
Section 2—Advertising		
Study website	High	Website live
Poster displays	High	Posters in non-PIC GP surgeries, local gyms and classes; hospital corridors; community centres
Local pharmacies	High	Posters displayed in pharmacies in Tesco (n = 26) and Sainsbury’s (n = 27) and local independent pharmacies
Large local employers and universities	High	21 companies and six universities advertised study via intranet
Press releases	Medium	Local newspapers printed two articles Item aired on local radio
Social media—Facebook and Twitter	Medium/ low	Accounts live and linked to SW pages and other relevant sites
Section 3—Other		
Alter emphasis to target route 1	High	Altered posters and recruitment drive to focus on route 1 only
Close monitoring of recruitment rates and monthly recruitment targets	High	Figures examined weekly
Research network support	High	Area specific strategies given to network staff to implement locally CLRN nurse in Trent trained and engaged with CLRN in South West England
Establish links with other health professionals	High to medium	TMG members presented at dietetics meetings as well as to gym managers and fitness club managers Specialist weight management clinic advertising study
Collaborate with other weight loss studies	Medium	Unsuccessful due to lack of studies.
Maximize use of flagging systems on practice databases	Medium	Unsuccessful due to complexities of various practice systems
Manage screening process and follow up of route 2 participants	Medium	Contact maintained with route 2 participants but emphasis that they must contact the study team with evidence of their weight loss
Attend and present at local health events	Low	2 events attended but little impact on recruitment
Section 4—actions not pursued (and reasons why)		
Use pharmacies as PICs	High	Not pursued due to resource implications involved in training sites
Complete database searches for PICs	High	Not pursued due to lack of REC approval
Increase visits to PICs to problem solve	Low	Not pursued due to lack of resource and likely low impact on recruitment rates
Link in with relevant patient groups	Low	Not pursued as study closed to recruitment
Target discussion forums/threads on the internet	Low	Not pursued as study closed to recruitment
Create links with other slimming groups	Low	Not pursued due to SW involvement
Placing adverts	Medium	Attempted to advertise on relevant internet sites but they were not appropriate/willing Paper, TV and radio—cost proved too expensive
Identify a local celebrity to champion the study	Medium	Attempted but unsuccessful

*GP* general practitioner, *SW* slimming world, *HQ* head quarters, *PIC* participant identification centre, *CLRN* comprehensive local research network, *REC* research ethics committee

#### High priorities

Independent verification of 5 % weight loss was creating a barrier to full recruitment, so high priority was given to targeting only those who were more likely to be able to provide this information easily. Extra PICs were recruited and trained to only approach those who fulfilled the criteria for recruitment via route 1. Making follow-up contact with route 2 individuals (n = 1043) was very resource intensive and led to few recruits (11.2 % of our total sample). The team stopped doing these follow-ups leaving it up to the individual if they wanted to get in touch with the study team once they had lost 5 %.

In an effort to sustain motivation of PIC staff, we increased use of newsletters and telephone contact [3], a relatively straightforward activity completed by the trial administrator. We also introduced a competition and financial incentives for exercise on referral and SW staff by for top recruiters (Table 3). This may be an effective strategy for future trials but feedback suggests that in this case, winning the ‘prize’ of top recruiter was not a high priority for most PIC staff.

We made SW high priority PICs over GP practices and exercise on referral as their clients proved to be most likely to have lost weight and, crucially, be able to provide evidence. The study team were also able to increase contact with SW and present the trial at more regional consultants’ monthly meetings as well as at various weekly clubs. Although this required a lot of researchers’ time and out of hours work, it was key to delivering the study to those who could recruit for us. Eleven regional meetings were attended with 84 consultants then a further 47 local meetings where the study team was able to present the trial to SW clients directly. It was found to be more effective to attend the consultants’ meetings where between five and ten consultants were present and the study team could speak directly about the study to a number of those who lead the classes (often several classes). Anecdotally, potential participants were more likely to respond to the consultants’ backing for the study than an unfamiliar member of the study team attempting to recruit them.

Advertising was also made high priority as widespread use of posters and email adverts was, low cost, time efficient and a potentially far reaching option [24]. Posters providing brief information about the study including eligibility criteria and contact details were placed in local GP practices and pharmacies—including those based in large supermarket chains. Along with adverts circulated to employees of large companies and universities in South Wales, this was a relatively simple approach to disseminating information to a large audience. The impact of this approach cannot be quantified, however a noticeable increase in approaches from interested individuals came as a response to the adverts. Future trials would benefit from drawing up an advertising strategy early on in their set up [24].

#### Medium priority

Utilising social media was also included in the implementation plan as a medium level priority. Time constraints however meant that it was not used in such a way as to specifically target potential participants, something which has been found effective [25], but rather utilised to increase the visibility of the study. Those linking in with the study via Facebook and Twitter tended to be other weight loss entities or health professionals rather than potential participants.

Other actions that were given medium level priority included establishing links with other health professionals. Our Trial Management Group members presented the trial at dietetics meetings as well as to gym managers and fitness club managers in an effort to get them to advertise the trial to their clients. We also advertised in specialist weight management clinics. These efforts did not translate into a noticeable impact on expressions of interest. Potential reasons for this being uncertainty or concern in relaying information to clients/patients [4].

#### Low priority

The strategy details a number of other actions that were planned but not implemented for various reasons (see Table 3). The study team and wider management group made every effort to address the issues being faced and challenges of recruiting participants.

In summary, the inclusion of a feasibility stage in the WILMA trial would have allowed for an assessment of the recruitment process, eligibility criteria and proposed routes (including a formal review process and associated timelines) and could have identified problems earlier and ensured resources were appropriately directed. Shifting our emphasis and resources to those who were eligible via route 1 for example proved the more effective strategy. We also identified a number of targeted advertising strategies which could have been accommodated in the budgeting stage of the application—something which future trials should consider. Social media offered access to a potentially huge audience who could have been targeted via adverts and offered incentives to take part. Other advertising strategies in this setting were also identified that could be implemented with minimal resource implications.

Development of a detailed recruitment strategy and implementation plan is recommended at the work up stage of future trials. Regular ongoing monitoring of those plans and strategies is crucial. A key learning point from our experience is that, as part of that recruitment strategy, careful consideration needs to be given to the viability of entry criteria at the outset. Careful thought about how to engage recruiters and keep them engaged needs to form part of this strategy and it needs to include the resources required to do this which may in fact be much more than anticipated. Keeping recruiters on board and motivated is resource intensive. Some consideration should also be given to engaging recruiters who have never been involved in research before and may not see the value or may have competing interests.

## Conclusions

Difficulties encountered in the WILMA trial highlight a number of key issues that could impact on the successful delivery of future community-based trials in this area. Some of these issues were beyond the control of the study team and can apply to trials generally, i.e., governance issues relating to costs and the approval process for recruiting via the NHS and potentially other health care systems. Based on our experience, we suggest that it is very important to (1) develop a detailed recruitment strategy early on, one which is carefully monitored and considers recruitment from different sources (if applicable), (2) not to underestimate the amount of time required to gain governance approvals, costs and network support in order to recruit participants as well as the impact this will have on the workload of the Trial Manager and study team and (3) conduct a feasibility study first. It is not sufficient to rely on previous experience of working in a particular setting as this is not guaranteed to translate to all future scenarios. In order to make recruitment strategies more evidence based, it is important to explore these avenues in a more structured way and potentially engage with government agencies, such as the DoH, as part of this. Not addressing these points risks having to invest large amounts of resource and time to resolve these problems.

This is a unique reflection of the challenges experienced during the delivery of one specific trial but it is hoped that, along with others experiences, future pragmatic RCTs in this area will be better informed and more equipped to deal with these challenges and thus more likely to complete according to plan, thereby ensuring that the research question is answered and the resources are well spent.

## Abbreviations

DoH: Department of Health
RCT: randomised controlled trials
MRC: medical research council
NHS: national health service
WILMA: weight loss maintenance in adults
BMI: body mass index
MI: motivational interviewing
HTA: health technology assessment
PIC: patient identification centre
GP: general practitioner
ETC: excess treatment cost
QOF: quality of outcomes framework
MIP: motivational interviewing practitioner
SW: slimming world
NISCHR: national institute of social care and health research
UKCRN: United Kingdom clinical research network
HRA: health research authority
R&D: research and development
